# COVID-19 versus Seasonal Influenza: Comparison of Profiles of Older Adults Hospitalized in a Short-Term Geriatric Ward in France

**DOI:** 10.4269/ajtmh.21-0480

**Published:** 2021-12-02

**Authors:** Lidvine Godaert, Agnès Cebille, Emeline Proye, Moustapha Dramé

**Affiliations:** ^1^ Department of Geriatrics, General Hospital of Valenciennes, Valenciennes, France;; ^2^ Faculty of Medicine, University of the French West Indies, Fort-de-France, Martinique, France;; ^3^ Department of Clinical Research and Innovation, University Hospital of Martinique, Fort-de-France, Martinique, France

## Abstract

The objective was to compare the profile and outcomes of older adults admitted to a geriatric short-stay unit for COVID-19, to those of older adults admitted to the same unit for seasonal influenza infection. This was an observational study performed in a General Hospital in France. Patients ≥ 70 years admitted to a geriatric short-stay unit for COVID-19 between March 18 and November 15, 2020 were included. They were compared with patients of the same age group, admitted to the same geriatric short-stay unit for seasonal influenza infection over the periods January to March 2019 and January to March 2020. Data collection included demographic information, medical history, clinical signs and symptoms, outcomes, and hospital discharge patterns. Descriptive and intergroup comparison analyses were performed. In total, 153 patients were included in the study, 82 in the seasonal influenza group, and 71 in the COVID-19 group. The average age was 87.6 ± 4.8 and 87.6 ± 6.5 years in the COVID-19 and seasonal influenza groups, respectively. There was no difference between groups regarding the Charlson comorbidity index (3.4 ± 3.0 versus 3.4 ± 2.8). The seasonal influenza group more often had fever, cough, sputum, and renal failure, whereas the COVID-19 group more often experienced diarrhea, and death. The COVID-19 group was frequently living in collective housing. The profile at admission of older adults hospitalized for COVID-19 or seasonal influenza infection was similar. Although fever and respiratory signs were less common in the COVID-19 group, these patients experienced more complications (such as renal failure or oxygen therapy requirement) and higher mortality.

## INTRODUCTION

Infections take a heavy toll on older adults every year,^1^ especially respiratory infections, whether viral or bacterial etiology.^2^^,^^3^ For example, severe clinical forms of seasonal influenza are responsible for high numbers of hospitalizations every year in older populations.^4^ At the beginning of 2020, the world was confronted with a surge in viral respiratory infections due to COVID-19.^5^ Rapidly, many studies were published describing the clinical and biological characteristics of patients affected by this new disease. The most frequently observed clinical signs in COVID-19 (fever, cough, asthenia, dyspnea . . .)^6^ are very similar to those observed in other respiratory viral diseases such as influenza or the syncytial virus.^7^^,^^8^ There are also similarities in the mode of transmission, respiratory complications, and the populations most vulnerable to severe forms of COVID-19. In both COVID-19 and influenza, age appears to be a risk factor for developing a severe form, and is associated with higher mortality.^9^^–^^11^

The objective of this study was to compare the profile of patients aged 70 years or older admitted to a short-stay geriatric unit for COVID-19 infection, with the profile of patients of the same age admitted to the same unit during the two previous influenza seasons. In a second step, we compared the outcomes of these two populations of infected older adults.

## METHODS

### Study design.

This was an observational longitudinal study performed in the General Hospital of Valenciennes (France). Data were recorded retrospectively using the hospital data-processing system. The study was performed in accordance with the Declaration of Helsinki and French legislation relating to research involving human subjects. The study received the approval of the Martinique Institutional Review Board (registered under the number 2020/044).

### Patient populations.

Patients aged 70 years or older admitted to the geriatric short-stay unit of Valenciennes General Hospital (France) for severe acute respiratory syndrome coronavirus 2 (SARS-CoV-2) infection (COVID-19) between March 18 and November 15, 2020, were included. They were compared with patients of the same age group, admitted to the same geriatric short-stay unit for seasonal influenza infection over the periods January to March 2019 and January to March 2020 (i.e., the previous two seasonal influenza epidemic periods).

Patients with concomitant COVID-19 and seasonal influenza infection, and patients who objected to the use of their data for research purposes were not included. Patients were considered to have COVID-19 when they had real-time polymerase chain reaction (RT-PCR) confirmed SARS-CoV-2, or when they had a clinical picture consistent with respiratory infection, associated with the following chest computed tomography (CT) imaging characteristics obtained after day 4 because the onset of symptoms: bilateral, multifocal, and peripheral ground glass pulmonary opacities with or without consolidation, or crazy paving, or air bronchogram, or reticular pattern. Patients were considered to have seasonal influenza when their RT-PCR influenza test were positive. Patients with asymptomatic forms of COVID-19 were excluded.

### Data collection.

Data collection included the type of respiratory infection, demographic information, medical history, clinical signs and symptoms, outcomes, and hospital discharge patterns. Demographic characteristics included age at admission, sex, and place of residence. History of falls as well as history of respiratory disease were noted. Presence in the medical files of dementia syndrome, dependency in Katz’s activities of daily living (ADL), and impaired mobility were recorded. Time since the onset of signs and symptoms, as well as the presence or absence of the following concurrent features were recorded: fever, cough, sputum, dyspnea, asthenia, diarrhea, delirium syndrome, and falls. Comorbidity burden was assessed using the Charlson comorbidity index. The number of medications per day was collected. Biological testing included blood count (neutrophils, lymphocytes, and thrombocytes), serum albumin and C-reactive protein levels, and alanine and aspartate aminotransferases (ALAT and ASAT). Renal function was explored using the glomerular filtration rate (GFR) rated by Cockcroft & Gault formula. Neutrophilia was defined by a neutrophil count greater than 10 × 10^9^/L. Lymphopenia was defined by a lymphocyte count under 1 × 10^9^/L. Thrombopenia was defined by a thrombocyte count under 150 × 10^9^/L. Nutritional status was explored using serum albumin level. Patients were divided into three groups according to their serum vitamin D level: replete group (> 30 ng/mL), insufficiency group (10–30 ng/mL), and deficiency group (< 10 ng/mL). Hypovitaminosis D was defined by a serum vitamin D level under 30 ng/mL. Patients were considered to have liver dysfunction when the level of ALAT or ASAT was more than twice the normal values. Renal failure was defined as a GFR rated by the Cockcroft & Gault formula under 60 mL/min. Use of oxygen therapy during hospitalization was recorded. Length of stay, and discharge patterns (death, discharge to home, or nursing home, or long-stay ward) were collected.

### Statistical analysis.

Quantitative variables are described as mean and SD (m ± SD), and categorical variables as number and percentage (*n*, %). Baseline characteristics were compared between groups using Student’s *t*-test or Mann–Whitney test, and the χ^2^ or Fisher exact test for categorical variables, as appropriate. Tests were considered significant for *P* values < 0.05. Statistical analyses were performed using SAS version 9.4 (SAS Institute Inc., Cary, NC).

## RESULTS

No patient objected to the use of his/her data. No patient experienced concomitant COVID-19 and seasonal influenza. In total, 153 patients were included in the study, 82 in the seasonal influenza group, and 71 in the COVID-19 group. The characteristics of the study population are presented in Table 1. Among the 71 COVID-19 patients, 69 had confirmed diagnosis by RT-PCR, and two by CT imaging. The average age was 87.6 ± 4.8 and 87.6 ± 6.5 years in the COVID-19 and seasonal influenza groups, respectively (*P* = 0.99). The time between the onset of signs/symptoms and hospital admission was not significantly different between groups (2.8 ± 3.3 versus 3.8 ± 4.2 days, seasonal influenza versus COVID-19, respectively; *P* = 0.15). The average number of medications was 7.1 ± 3.0 in the seasonal influenza group versus 7.2 ± 3.7 in the COVID-19 group (*P* = 0.87). No difference was found between groups regarding the Charlson comorbidity index (3.4 ± 3.0 versus 3.4 ± 2.8; *P* = 0.96), or serum vitamin D level (23.5 ± 12.6 versus 25.5 ± 11.7; *P* = 0.35), or serum albumin level (33.6 ± 4.0 versus 32.5 ± 3.8; *P* = 0.10). There were also no statistical differences between the three groups of vitamin D. Length of stay was similar between groups (9.4 ± 6.2 days in the influenza group versus 9.8 ± 6.6 days in the COVID-19 group; *P* = 0.74). As indicated in Table 2, the seasonal influenza group more often had fever, cough, sputum, and renal failure, whereas the COVID-19 group more often experienced diarrhea, and death. The COVID-19 group were frequently living in collective housing (i.e., sheltered housing or nursing home). The comparison of the other characteristics between the two groups is presented in Table 2.

**Table 1:** Characteristics of the study population (*N* = 153)

Variables	*n*	%
Type of infection	–	–
Seasonal influenza	82	53.6
SARS-CoV-2 infection (COVID-19)	71	46.4
Female sex	102	66.7
Age ≥ 85 years	109	71.2
Living in collective housing (sheltered housing or nursing home)	62	40.5
History of falls	72	47.1
History of respiratory diseases	44	28.8
Dementia syndrome	90	58.8
Dependent for ADLs	130	85.0
Impaired mobility	91	59.5
Serum albumin level (m ± SD)	144	33.1 ± 4.0
Serum CRP level (median ± IQR)	153	59 ± 106
Vitamin D groups	–	–
Replete (Serum vitamin D > 30 ng/mL)	38	28.8
Insufficiency (Serum vitamin D 10–30 ng/mL)	74	56.1
Deficiency (Serum vitamin D < 10 ng/mL)	20	15.1
Concurrent signs/symptoms	–	–
Fever	104	68.0
Cough	86	56.2
Sputum	25	16.3
Dyspnea	104	68.0
Asthenia	103	67.3
Diarrhea	21	16.7
Delirium	38	24.8
Falls	42	27.5
Neutrophilia (> 10 × 10^9^/L)	47	30.7
Lymphopenia (< 10^9^/L)	100	65.4
Thrombopenia (< 150 × 10^9^/L)	48	31.4
Renal failure (GFR < 60 mL/min)	68	44.4
Liver dysfunction	50	41.3
Oxygen therapy	101	66.0
Discharge patterns	–	–
Discharged to home	90	58.8
Discharged to geriatric rehabilitation unit	24	15.7
Discharged to other medical wards	1	0.7
Death	38	24.8

ADL = activities of daily living; CRP = C-reactive protein; GFR = glomerular filtration rate; m ± SD = mean ± SD; IQR = interquartile range; SARS-CoV-2 = severe acute respiratory syndrome coronavirus 2. Missing values: Liver dysfunction (*n* = 32); serum albumin level (*n* = 9); vitamin D groups (*n* = 21).

**Table 2:** Comparison between patients with seasonal influenza and patients with COVID-19

	Seasonal influenza		COVID-19		
Variables	*N* = 82		*N* = 71		
	*n*	%	*n*	%	*P*
Female sex	52	63.4	50	70.4	0.36
Age ≥ 85 years	60	73.2	49	69.0	0.57
Living in collective housing (sheltered housing or nursing home)	24	29.3	38	53.5	0.002
History of falls	30	36.6	42	59.2	0.005
History of respiratory diseases	27	32.9	17	23.9	0.22
Dementia syndrome	48	58.5	42	59.2	0.94
Dependent for the ADLs	71	86.6	59	83.1	0.55
Impaired mobility	52	63.4	39	54.9	0.29
Serum albumin level (m ± SD)	81	33.6 ± 4.0	63	32.5 ± 3.8	0.10
Serum CRP level (median ± IQR)	82	53 ± 88	71	84 ± 110	0.28
Hypovitaminosis D (serum vitamin D level < 30 ng/mL)	52	69.3	38	66.7	0.74
Concurrent signs/symptoms					
Fever	63	76.8	41	57.8	0.01
Cough	66	80.5	20	28.2	< 0.0001
Sputum	23	28.1	2	2.8	< 0.0001
Dyspnea	59	72.0	45	63.4	0.26
Asthenia	57	69.5	46	64.8	0.53
Diarrhea	6	7.3	15	21.1	0.01
Delirium	19	23.2	19	26.8	0.61
Falls	20	24.4	22	31.0	0.36
Neutrophilia (> 10 × 10^9^/L)	25	30.5	22	31.0	0.95
Lymphopenia (< 1 × 10^9^/L)	53	64.3	47	66.2	0.84
Thrombopenia (< 150 × 10^9^/L)	31	37.8	17	23.9	0.06
Renal failure (GFR < 60 mL/min)	47	57.3	21	29.6	0.0006
Liver dysfunction*	26	41.3	24	41.4	0.99
Oxygen therapy	47	57.3	54	76.1	0.02
Discharged to home	60	73.2	30	42.3	0.0001
Deaths	10	12.2	28	39.4	0.0001

ADL = activities of daily living; CRP = C-reactive protein; GFR = glomerular filtration rate, rated by Cockcroft & Gault formula m ± SD = mean ± SD; IQR = interquartile range.

* Liver dysfunction: level of alanine aminotransferase (ALAT) or aspartate aminotransferase (ASAT) more than twice the normal value.

## DISCUSSION

We compared the clinical and biological profile of 153 hospitalized patients aged 70 years or older, namely 82 with seasonal influenza and 71 with COVID-19. In our study, older patients hospitalized with COVID-19 or seasonal influenza had the same characteristics in terms of sex, age, dependency, comorbidities (including respiratory and cognitive), and number of medications taken. These findings would indicate that in an aged population, the same people are experiencing moderate to severe forms of seasonal influenza and COVID-19. Burn et al. studied the phenotype of adult patients hospitalized with COVID-19 compared with adult hospitalized for seasonal influenza, and found that those hospitalized for COVID-19 had comparable or lower prevalence of comorbidities compared with those with seasonal influenza.^12^ In our study, subjects with COVID-19 more frequently self-reported having had at least one fall in the previous year, compared with patients with influenza (59.2% versus 36.6%; *P* = 0.005). Falls have often been identified as markers of frailty in older persons. This suggests that older subjects admitted to hospital for COVID-19 may be frailer than those hospitalized for seasonal influenza.

In this study, we noted that older adults hospitalized for COVID-19 more frequently lived in a nursing home or in long-term care facilities (53.5% versus 29.3%; *P* = 0.002). This observation could suggest a greater level of contagiousness of COVID-19 compared with seasonal influenza. However, there are other potential explanations for this observation. First of all, vaccination is available to prevent seasonal influenza, although the effectiveness of influenza vaccination in preventing the complications of infection (severe forms, hospitalization, and death), particularly in nursing homes or long-care facilities, remains unclear.^13^ Nevertheless, influenza vaccine coverage in nursing homes is reportedly quite good in France.^14^ This is likely to help reduce the circulation of the virus in nursing homes or long-care facilities. Secondly, COVID-19 is a new disease, and the pandemic context probably favored the transfer to hospital of individuals with suspected or confirmed infection. This was particularly true during the “first wave” of COVID-19 (approximately between March and June 2020). Indeed, at the start of the pandemic, patients were often hospitalized as soon as a diagnosis of COVID-19 infection was made. In nursing homes, screening was facilitated by systematic screening campaigns in healthcare institutions. However, in the “second wave” (approximately between September and November 2020), hospitalization instructions had changed, and patients with mild or moderate forms of COVID-19, living in nursing homes, were kept in nursing home. Only the most at-risk or severe cases were hospitalized during the second wave. In our study, 23 (60.5%) patients infected by COVID-19, and living in a nursing home were included during the “first wave” and 15 (39.5%) during the “second wave.”

The clinical signs or symptoms observed at admission differed between patients hospitalized for COVID-19 and those admitted for seasonal influenza in our study. Fever was significantly more common in older patients with seasonal influenza (76.8% versus 57.8%, *P* = 0.01). Qu et al reported similar findings in a younger adult population.^15^ Fever is a common sign in infectious disease and is frequently observed in both COVID-19 and seasonal influenza. In our study, 57.8% of patients with COVID-19 had fever. This is lower than the rates observed by other authors in younger populations.^6^^,^^16^^,^^17^ Guan et al reported fever in 88.7% of patients (median age: 47 years) with COVID-19 during hospitalization and 43.8% on admission.^6^ Other authors have studied the clinical presentation of COVID-19 in older populations.^6^^,^^9^^,^^11^^,^^16^^,^^18^^–^^20^ Fever seems to be less frequent in older populations than in younger adults. The finding that older subjects less frequently present fever during infection is common.^21^ In patients with COVID-19 in our study, fewer than 60% had fever. Our population is older (mean age 87.6 ± 6.5 years) than in most other reports to date of COVID-19 presentation in older adults.^10^^,^^11^^,^^18^ Conversely, 76.8% of patients with influenza had fever in our study. Masse et al studied the epidemiology of respiratory pathogens among nursing home residents with acute respiratory infections, and reported that fever was more common in seasonal influenza than in other types of acute respiratory infection, especially respiratory viruses other than influenza. Cough and sputum were also more common in patients with seasonal influenza in our study (cough: 80.5% versus 29.8%, *P* < 0.0001; sputum 28.0% versus 4.3%, *P* = 0.001).^7^ Souty et al studied the most common symptoms/signs of several viral infections, including influenza, and found that cough and absence of dyspnea were significantly associated with seasonal influenza.^8^ Mosnier et al also observed that coughing is reported by more than 90% of older adults with influenza.^22^ Coughing seems to be less common in people with COVID-19, with rates ranging from 33.3% to 82.6% across studies.^6^^,^^16^^,^^18^^,^^19^^,^^20^^,^^23^^,^^24^ Most authors did not observe a difference in the prevalence of cough according to age in COVID-19 subjects.^10^^,^^11^ In our study, coughing in older adults with COVID-19 was less common than in other studies with younger COVID-19 patients.^6^^,^^10^ We cannot exclude the possibility that this clinical sign was underdiagnosed. Nevertheless, Liu et al observed coughing in only 33.3% of people aged 60 years or older (*N* = 18).^11^ In our study, coughing was present in 28.2% of people aged 70 years or older with COVID-19 (*N* = 71).

We observed that diarrhea was significantly more frequent in people with COVID-19 compared with patients with seasonal influenza (21.1% versus 7.3%; *P* = 0.01). Diarrhea is present in COVID-19 at a variable frequency according to different authors (from 2% to 35.5%).^6^^,^^10^^,^^16^^,^^18^^–^^20^^,^^23^^,^^25^ Pan et al observed that people infected by COVID-19 with digestive symptoms arrive at the hospital later.^26^ Liang et al suggested that the prevalence of diarrhea may be underestimated in particular because the diagnostic criteria could be different between studies.^27^ The prevalence of diarrhea appears to be higher in older subjects, ranging from 11.9% to 35.5%.^18^^,^^19^^,^^23^^,^^25^ SARS-CoV-2 is excreted in the stool in about one in three patients.^28^ The hypotheses put forward to explain the tropism of COVID-19 for the intestine remain unclear. Some authors have suggested a link with the human angiotensin converting enzyme 2 receptor.^26^^,^^27^ Inflammatory response during infection by SARS-CoV-2, and cytokine storm may also be involved.

With regard to complications, in our study, the use of oxygen therapy was more frequent in patients with COVID-19 than in those with seasonal influenza (76.1% versus 57.3%; *P* = 0.02). Acute respiratory distress seems to be more frequent in COVID-19 than in seasonal influenza.^29^ The most frequent complication of COVID-19 is respiratory distress syndrome,^30^ frequently requiring hospitalization, and it is associated with high mortality.^17^^,^^24^ Respiratory distress syndrome is preceded by dyspnea and occurs on average 8 to 10 days after the first signs.^16^ Its etiology is currently linked to the inflammatory phenomenon termed “cytokine storm.”^31^ An adequate innate immune response is the first line of defense against viral infection. The cytokine storm can be defined as an excessive immune response characterized in particular by high levels of proinflammatory cytokines (e.g., Interleukin (IL)-6, IL-1β, tumor necrosis factor).^32^ Vitamin D has been mentioned by some authors as being involved in the regulation of the immune response.^33^ Vitamin D deficiency could be associated with adverse outcomes in COVID-19.^34^^,^^35^ In our study, nearly 70% of patients had vitamin D levels below 30 ng/mL. Dramé et al. published a systematic review that suggests an association between vitamin D deficiency and risk of COVID-19 in aged people.^36^ In addition, vitamin D deficiency appears to expose these subjects to a greater risk of adverse outcomes.

In seasonal influenza, bacterial pneumonia is the most common complication.^37^ The cytokine storm has not been documented in this viral infection, except during the pandemic of H1N1 influenza A.^38^ Nevertheless, vitamin D has also been mentioned as a mean to reduce case fatality rates in seasonal influenza by reducing secondary bacterial infections.^39^ Sabetta et al. suggested that vitamin D deficiency is associated with higher prevalence and longer duration of seasonal influenza.^40^ The efficacy of chronic supplementation in vitamin D has not been clearly demonstrated.^41^^,^^42^ It is possible that the chosen target level is different for infectious risk than for bone risk.^43^

In our study, mortality was higher in patients with COVID-19 compared with those with seasonal influenza (39.4% versus 12.2%; *P* = 0.0001). Cates et al made a similar observation in a population with a median age of 70 years.^29^ In both seasonal influenza and COVID-19, old age is a risk factor for adverse outcomes and mortality.^9^^,^^17^^,^^37^ Some authors have suggested a higher risk of cytokine storm in older people, notably due to the phenomenon termed “inflamm-aging,” namely a chronic state of low-level inflammation due to aging.^44^ Older adults also often suffer from comorbidities that are associated with higher mortality in these infections.^18^^,^^30^^,^^37^ Nevertheless, mortality in COVID-19 is higher than in patients with influenza.^45^^,^^46^ Vaccination against seasonal influenza could contribute to reducing the risk of complications and death.^47^ Furthermore, therapeutic management for seasonal influenza is well known and there are effective antiviral agents available that have proven their effectiveness in reducing the risk of complications and death.^48^ These treatments, however, have not been shown to be effective against COVID-19.^49^ The use of corticosteroids in COVID-19 during respiratory complications appears to reduce mortality,^50^ but this approach was not recommended at the time of the first wave of COVID-19 in France.

Our study has some limitations. It included only patients who were hospitalized, and thus, generalizability to the whole population of older adults infected with either seasonal influenza or COVID-19 is not warranted. The number of patients included was relatively limited, particularly for patients with seasonal influenza. We cannot exclude the possibility of recruitment bias among the patients with COVID-19, since during the first wave (March to June 2020), patients with confirmed infection were frequently admitted to hospital, regardless of the severity of their clinical state. Nevertheless, the majority of patients admitted to geriatrics units dedicated to management of older COVID-19 patients had multiple pathologies, and frailty, whereas patients with asymptomatic forms were excluded. During the second wave, management practices had changed, and only patients with severe forms of COVID-19 were admitted to hospital. Furthermore, data collection was retrospective, although there was a very low rate of missing data (except regarding liver function). Our study also has some strengths. It is the first study to compare the clinical profile at admission of patients older than 70 years hospitalized for severe forms of seasonal influenza or COVID-19. Patients were also admitted to specialized geriatrics units, or geriatrics units dedicated to COVID-19 care, thus guaranteeing better quality data recording as regards the clinical signs noted in the medical files, because patients were cared by physicians specialized in geriatrics.

In conclusion, in this study, the clinical and biological profile at admission of older adults hospitalized for COVID-19 or seasonal influenza was similar. Although fever and respiratory signs were less common in patients infected with COVID-19, these patients nonetheless experienced more complications and higher mortality. These findings plead in favor of the greater severity of COVID-19 infection, and call for more aggressive management. Indeed, it is a novel coronavirus and the immune response it provokes differs from that observed in other viral respiratory infections. Wide vaccine coverage against SARS-CoV-2, associated with management strategies targeting the cytokine storm, could help to improve the outcomes in older subjects with COVID-19.
